# Sentinel Node Biopsy in Laryngeal Cancer: A Systematic Review and Meta-Analysis

**DOI:** 10.3390/diagnostics15030366

**Published:** 2025-02-04

**Authors:** Pegah Sahafi, Ramin Sadeghi, Emran Askari, Azadeh Sahebkari, Mitra Ghahraman, Ehsan Khadivi, Kamran Khazaeni, Vahid Reza Dabbagh Kakhki, Sara Harsini

**Affiliations:** 1 Nuclear Medicine Research Center, Mashhad University of Medical Sciences, Mashhad 9176699199, Iran; sahhafip4001@mums.ac.ir (P.S.); sadeghir@mums.ac.ir (R.S.); emran.a69@gmail.com (E.A.); sahebkari.azadeh@gmail.com (A.S.); ghahremanm4001@mums.ac.ir (M.G.); dabbaghvr@mums.ac.ir (V.R.D.K.); 2 Sinus and Surgical Endoscopic Research Center, Mashhad University of Medical Sciences, Mashhad 9176699199, Iran; khadivie@mums.ac.ir (E.K.); khazaenik@mums.ac.ir (K.K.); 3 Department of Molecular Imaging and Therapy, BC Cancer Research Institute, Vancouver, BC V5Z 1L3, Canada

**Keywords:** sentinel lymph node, laryngeal squamous cell carcinoma, larynx, SCC

## Abstract

**Background:** Sentinel lymph node (SLN) biopsy offers a minimally invasive approach to staging lymph node involvement in laryngeal squamous cell carcinoma (SCC). Despite its adoption in other cancers, its accuracy in laryngeal SCC remains under investigation. This systematic review and meta-analysis evaluates the diagnostic performance of SLN mapping in laryngeal cancer. **Methods:** A systematic search of MEDLINE, Scopus, and Google Scholar was conducted using the keywords “(larynx OR laryngeal) AND sentinel”, with no date or language restrictions. Studies reporting SLN detection rates and/or sensitivity in laryngeal SCC were included. A random-effects model was applied for data pooling, and subgroup analyses were performed based on tumor location (supraglottic versus transglottic) and mapping material (radiotracer versus blue dye). Publication bias was assessed using funnel plots and statistical methods. **Results:** Nineteen studies, encompassing 366 patients, were analyzed. The overall pooled SLN detection rate was 90.8% (95% CI: 86–94.1), and sensitivity was 88% (95% CI: 81–94). Supraglottic tumors demonstrated superior outcomes (detection rate: 93.7%, sensitivity: 96%) compared to transglottic tumors (detection rate: 84.7%, sensitivity: 71%). Radiotracers significantly outperformed blue dye, with detection rates of 90.8% versus 81.5% and sensitivities of 88% versus 77%. **Conclusions:** SLN mapping is a reliable technique for staging laryngeal SCC, particularly for supraglottic tumors, where high detection rates and sensitivity were observed. Radiotracers offer superior performance compared to blue dye, underscoring their clinical value. These findings support the feasibility and accuracy of SLN biopsy in laryngeal cancer, while emphasizing the importance of tumor location and mapping material.

## 1. Introduction

Laryngeal cancer is among the most prevalent malignancies of the respiratory tract, with cervical lymph node metastases significantly influencing survival outcomes in laryngeal squamous cell carcinoma (SCC) [1,2,3]. About 50% of laryngeal cancer patients survive 7 years; survival varies by location, with glottic highest (84%) and supraglottic lowest [4,5]. Despite advances in imaging modalities, detecting metastatic lymph nodes remains a considerable challenge. Occult lymph node metastases are commonly observed in head and neck SCCs, particularly in supraglottic laryngeal carcinomas, due to the dense lymphatic network in this region [6]. While early-stage glottic tumors exhibit a low risk of cervical lymph node metastasis (0–10%), advanced-stage tumors have a higher incidence, ranging from 10% to 35% [7]. Lymph node metastases occur at a very low rate in T1–T2 stages and in well-differentiated tumors [8].

Managing the clinically node-negative (N0) neck in head and neck cancer has been a subject of debate for decades. Most treatment centers favor elective neck treatment—either through surgery or radiation—when the risk of occult metastases surpasses 15% to 20%. However, this approach may lead to overtreatment in a substantial proportion of patients [6,9]. In supraglottic and advanced T-stage glottic laryngeal SCC, where no lymph node involvement is evident upon examination and imaging, the standard procedure typically involves bilateral elective neck dissection (END) [10]. The prevalence of occult metastases is notably higher in supraglottic tumors (23%), exceeding that of glottic tumors by 10.8%. Furthermore, advanced T-stage tumors are significantly more likely to develop occult metastases compared to early T-stage tumors [9]. A systematic review revealed that even in low T-stage supraglottic tumors (T1–T2), the rate of occult lymph node metastases was 18.4% [1].

Advanced imaging techniques, such as fluorine-18 fluorodeoxyglucose positron emission tomography/computed tomography (F-18 FDG PET/CT), have demonstrated moderate specificity but limited sensitivity for detecting cervical lymph node metastases in clinically N0 head and neck SCC (HNSCC) patients [11]. Consequently, there remains a critical need for less invasive, yet reliable, approaches for staging regional lymph nodes in laryngeal carcinoma.

Sentinel lymph node mapping has emerged as a promising method for detecting lymph node metastases, with growing evidence supporting its utility in laryngeal carcinoma [6,12]. Sentinel lymph node biopsy (SLNB) operates on the principle that cancer metastasizes in a predictable pattern through the lymphatic system, initially involving sentinel lymph nodes (SLNs) before spreading to other nodes. Thus, SLN status serves as a reliable indicator of regional lymphatic involvement [13,14,15]. Accurate detection of metastases relies on advanced techniques such as step serial sectioning and immunohistochemistry [16].

Despite its potential, SLNB is not without limitations. For instance, PET/CT scans, while offering good specificity and positive predictive value, lack sufficient sensitivity and negative predictive value, making them unreliable for excluding neck dissection in salvage laryngectomy for recurrent laryngeal SCC [17]. Given these considerations, we conducted an extensive systematic review and meta-analysis to evaluate the accuracy and success of SLN biopsy in staging cervical lymph node involvement in laryngeal SCC. This analysis aims to provide robust evidence on the feasibility and clinical utility of this technique.

## 2. Materials and Methods

### 2.1. Search Strategy and Inclusion Criteria

The systematic review was conducted in accordance with the PRISMA protocol [18], structured around the following PICO framework: PICO Patient—patients with proven laryngeal cancer who are candidates for laryngectomy and lymph node dissection; Intervention—sentinel node biopsy; Comparison—cervical lymph node dissection; Outcome—false negative rate.

Comprehensive searches of Medline, Scopus, and Google Scholar were conducted using the keywords: (larynx OR laryngeal) AND sentinel. No restrictions on language or publication date were applied. Only studies focused on SLN mapping and biopsy in SCC of the larynx were included. To ensure thorough coverage, reference lists and citing articles of the retrieved studies were examined for additional relevant studies. The last search was completed in March 2024.

Eligibility criteria required that studies report sufficient data to extract the detection rate and/or false-negative rate of SLN mapping in laryngeal SCC. Database searches and study selection were conducted independently by two authors. Discrepancies between the two reviewers were resolved through consultation with a third author. In cases of duplicate studies, only the most recent or comprehensive publication was included.

### 2.2. Quality Assessment

The quality of the included studies was evaluated using the Oxford Center for Evidence-Based Medicine checklist for diagnostic studies [19]. This checklist assesses several key areas:

Patient recruitment: Prospective consecutive inclusion was considered the optimal recruitment method.

Reference standard: The preferred reference standard was radical neck dissection for all patients, regardless of SLNB pathology results. Alternatively, long-term follow-up of patients with pathologically negative sentinel nodes was acceptable as a gold standard. It was critical that the reference standard be applied blindly to SLNB results.

Spectrum of patients: An ideal patient spectrum included all clinically node-negative (cN0) laryngeal cancer patients.

### 2.3. Statistical Analysis

Meta-analyses of sensitivity and detection rate were performed using a random-effects model (Der-Simonian and Laird method) [20]. This approach accounts for variability between studies, making it particularly suitable for pooling data across studies with heterogeneous characteristics. Heterogeneity was assessed using the Cochrane Q test, with significance set at *p* < 0.05, and quantified using the I^2^ index, which measures variability attributable to true heterogeneity rather than sampling error [21].

Publication bias was evaluated using funnel plots, Egger’s regression intercept, and Duval–Tweedie’s trim and fill method [22,23]. Funnel plots display the standard errors of included studies on one axis and their effect sizes on the other. Funnel plot asymmetry suggests potential publication bias, which was quantified using Egger’s regression intercept (*p* < 0.05 indicating significant bias). Duval–Tweedie’s trim and fill method adjusts for bias by iteratively removing smaller studies to achieve symmetry in the funnel plot, yielding an adjusted pooled effect size to estimate the impact of publication bias.

Meta-analytical calculations were performed using Comprehensive Meta-Analysis (version 2) and Meta-DiSc (version 1.4) [24]. Key diagnostic indices, detection rate, and false-negative rate were defined as follows:

Detection rate: The proportion of patients in whom at least one SLN was successfully identified.

False-negative rate: The proportion of patients with pathologically involved non-sentinel nodes despite having pathologically negative SLNs.

Subgroup analyses were conducted to explore the impact of lymphatic mapping materials (blue dye versus radiotracer) and tumor anatomical location (supraglottic versus transglottic) on outcomes.

The sensitivity and false negative rate of the sentinel node biopsy were reported based on elective cervical lymph node dissection pathology reports. There was no follow up report or any prognostic data in the included studies.

## 3. Results

**Figure 1:**
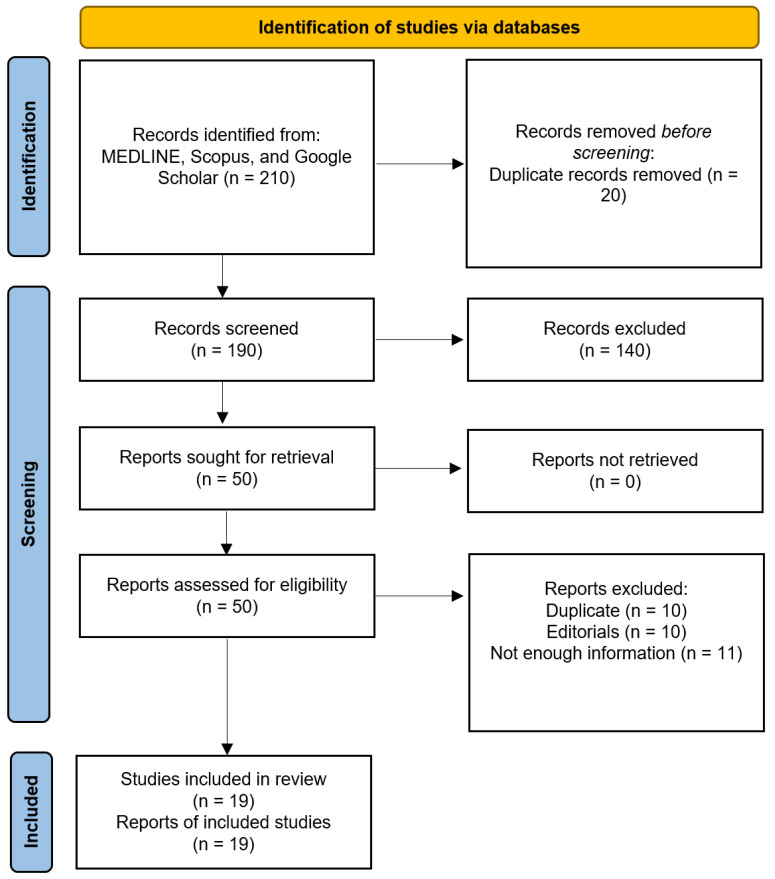
PRISMA flow diagram.

Figure 1 shows a summary of the search strategy and inclusion process of the study. Overall, 19 studies were included in the systematic review [12,25,26,27,28,29,30,31,32,33,34,35,36,37,38,39,40,41,42]. Among these studies, 13 provided data for separate subgroups of supraglottic and transglottic tumors. Three studies did not report the tumor T-stages, while the remaining studies included patients across T1 to T4 stages, comprising 39 T1, 129 T2, 106 T3, and 39 T4 cases. Additionally, two patients had a history of recurrent disease. A detailed summary of the included studies is presented in Table 1.

### 3.1. Detection Rate

**Figure 2:**
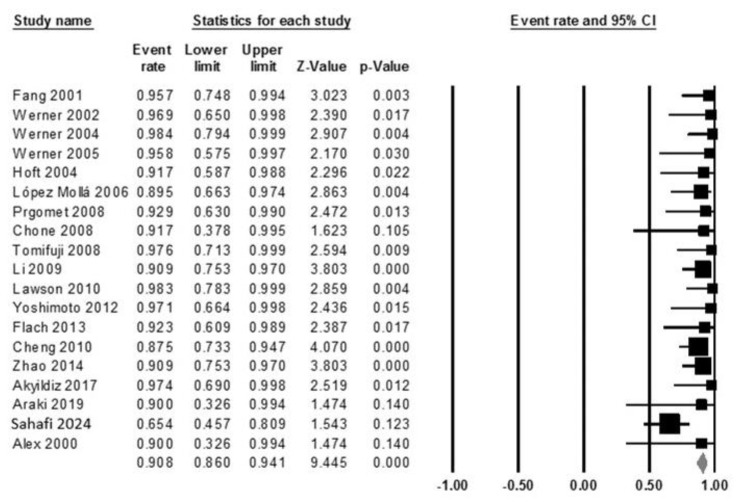
Forest plot illustrating the pooled detection rate across included studies.

The pooled detection rate for SLNB was 90.8% (95% CI: 86–94.1), as shown in the forest plot in Figure 2. Heterogeneity analysis indicated a Cochrane Q value of 23.02 (*p* = 0.189) and an I^2^ index of 21.80%, suggesting low variability among studies.

Subgroup analysis demonstrated differences in detection rates based on the SLN mapping method and tumor location. For SLN mapping materials, studies employing blue dye injection reported a pooled detection rate of 81.5% (95% CI: 67.8–90.2), whereas those using radiotracer injection achieved a significantly higher pooled detection rate of 90.8% (95% CI: 85.5–94.3). Tumor location also influenced the detection rate, with transglottic tumors exhibiting a pooled detection rate of 84.7% (95% CI: 75.9–90.7) compared to supraglottic tumors, which had a pooled detection rate of 93.7% (95% CI: 88.9–96.5). The majority of sentinel nodes were localized in cervical levels II and III, although three studies did not report sentinel node locations.

### 3.2. Sensitivity

**Figure 3:**
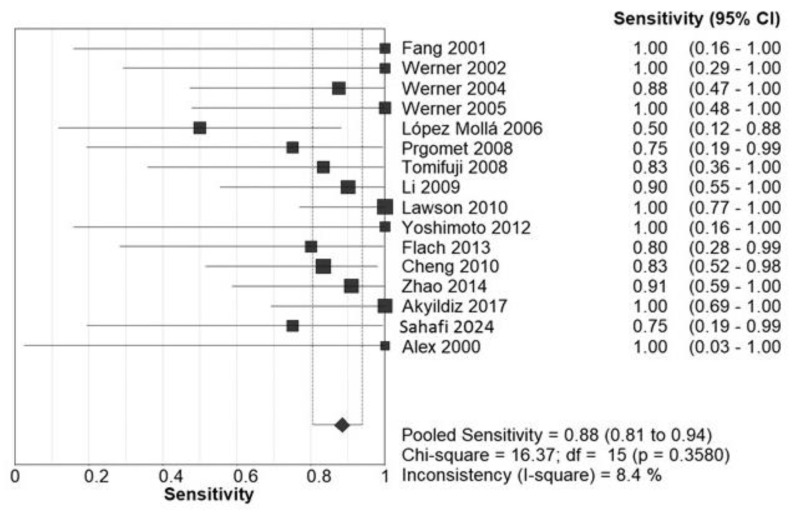
Forest plot illustrating the pooled sensitivity across included studies.

The pooled sensitivity for SLNB was 88% (95% CI: 81–94), as illustrated in the forest plot in Figure 3. Heterogeneity analysis revealed a Cochrane Q value of 36.2 (*p* = 0.0006) and an I^2^ index of 8.4%, indicating low heterogeneity.

Subgroup analyses for sensitivity highlighted differences based on mapping materials and tumor location. Blue dye injection yielded a pooled sensitivity of 77% (95% CI: 55–92), while radiotracer injection demonstrated superior sensitivity at 88% (95% CI: 80–94). Tumor location further impacted sensitivity outcomes, with supraglottic tumors showing a pooled sensitivity of 96% (95% CI: 88–99), significantly higher than transglottic tumors, which had a pooled sensitivity of 71% (95% CI: 52–86).

### 3.3. Quality Assessment

The quality of the included studies was evaluated using the Oxford Center for Evidence-Based Medicine checklist for diagnostic studies. Table 2 provides a detailed summary of the assessment, indicating that most studies adhered to high standards in patient recruitment, with consecutive or randomized inclusion, and robust application of reference standards, such as radical neck dissection. However, variability was observed in study design, particularly in the spectrum of patients and the completeness of blinding in reference standard application.

### 3.4. Publication Bias Evaluation

**Figure 4:**
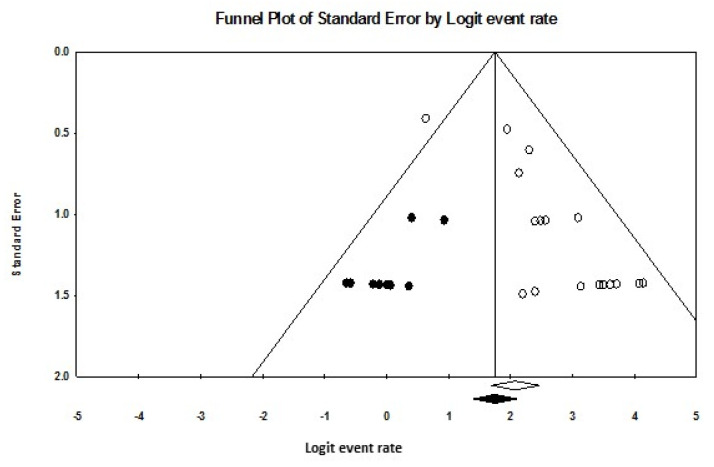
Funnel plot of detection rate pooling. White circles represent the included studies, and white diamond indicates the pooled detection rate of these studies. Black circles represent the studies trimmed to correct for asymmetry, while the black diamond represents the adjusted pooled effect size accounting for potential publication bias, as calculated using the Duval–Tweedie trim and fill method.

Publication bias was assessed through funnel plots and statistical methods. For detection rate, the funnel plot shown in Figure 4 displayed asymmetry, with Egger’s regression intercept calculated at 1.95 (*p* = 0.00006). After applying Duval–Tweedie’s trim and fill method and removing nine studies, the funnel plot achieved symmetry, resulting in an adjusted pooled detection rate of 81.8% (95% CI: 80.5–91.2), reflecting a 4% reduction from the original pooled rate.

**Figure 5:**
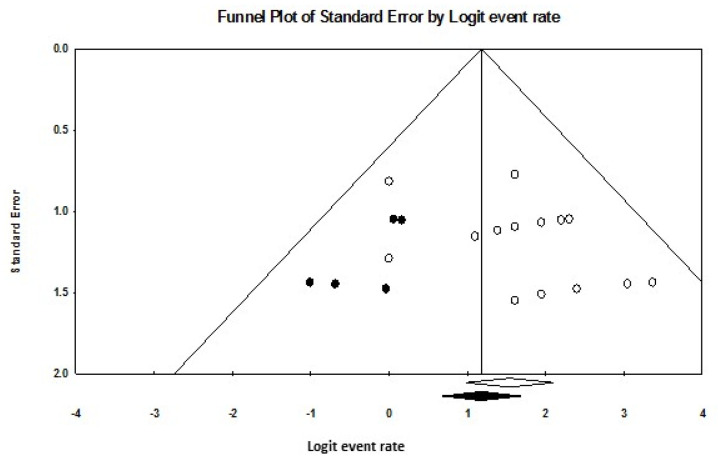
Funnel plot of sensitivity pooling. White circles represent the included studies, and white diamond indicates the pooled detection rate of these studies. Black circles indicate the studies trimmed to correct for asymmetry, with the black diamond representing the adjusted pooled effect size accounting for potential publication bias, as calculated using the Duval–Tweedie trim and fill method.

For sensitivity, the funnel plot depicted in Figure 5 also showed asymmetry, with Egger’s regression intercept at 1.50 (*p* = 0.051). Following the removal of five studies through the trim and fill method, a symmetrical funnel plot was obtained. The adjusted pooled sensitivity was 76.4% (95% CI: 66.4–84.2), reflecting an 8% reduction from the observed pooled sensitivity.

## 4. Discussion

About 50% of people with laryngeal cancer survive at least 7 years, with 31% living 10 years or longer. Survival rates depend on cancer location and spread. Glottis cancer has the highest survival rate, with a 5-year survival of 84% if localized. Supraglottic cancer has the lowest survival rates, around 30–61% [4,5].

At present, SLNB is a well established approach for evaluating lymph node involvement in certain cancer types, including breast cancer, melanoma, and select urogenital cancers [43,44,45,46]. Ongoing research continues to explore the potential application of this technique in other malignancies [47]. 

In the context of laryngeal cancer, the use of SLNB remains a topic of debate due to the intricate anatomy of the larynx and its complex network of lymphatic drainage pathways through the neck [48,49]. The presence or absence of cervical lymph node involvement is a critical factor that significantly impacts the staging and prognosis of laryngeal cancer.

Anatomical considerations also play a key role in understanding the distribution pattern of lymph node metastasis, with levels II and III being the most commonly affected regions [6].

While imaging modalities such as CT, MRI, and PET-CT provide valuable insights into cervical nodal disease and systemic metastases, they have limitations in reliably excluding metastases in clinically node-negative (N0) necks [17].

Additionally, SLNB shows considerable promise as a technique for staging cervical lymph nodes [12,25]. The findings from our meta-analysis highlight its potential as a reliable diagnostic tool, especially given the high pooled detection rate and sensitivity reported. Our study identifies key parameters in laryngeal cancers: mapping material (blue dye vs. radiotracer) and tumor location (supraglottic vs. infraglottic). These factors are crucial in diagnosis and may influence the risk of false-negative results in sentinel lymph node biopsy. These results support the adoption of SLNB as an alternative to more invasive procedures for staging cervical lymph node involvement in patients with laryngeal SCC.

While imaging modalities such as CT, MRI, and PET-CT provide valuable information about cervical nodal disease and systemic metastases, they fall short in reliably excluding metastases in clinically node-negative (N0) necks (38). In this context, SLNB offers a promising alternative, and our meta-analysis provides strong evidence of its diagnostic accuracy in laryngeal SCC.

This systematic review demonstrated pooled detection rates and sensitivity for SLNB of 90.8% and 88%, respectively, with low heterogeneity across studies (I^2^ indices of 21.8% and 8.4%). These findings affirm the reliability of SLNB in assessing lymph node involvement. Additionally, further analysis of diagnostic performance revealed significant variations based on mapping techniques and tumor location.

Radiotracer injection emerged as the superior mapping method, achieving significantly higher detection rates (90.8%) and sensitivity (88%) compared to blue dye injection (81.5% and 77%, respectively). These results are consistent with previous studies and highlight the advantages of radiotracers in identifying sentinel nodes that are deeply embedded in tissue layers, where blue dye may be less effective [12,27,50]. Additionally, accidental damage to the lymphatic vessels can result in the extravasation of blue dye, which not only impairs the overall visibility of the surgical field but may also delay wound healing [27]. Moreover, some patients have experienced allergic or anaphylactic reactions to blue dye [51]. The choice of tracer material is thus a critical factor in optimizing SLNB outcomes and should be carefully considered in clinical practice.

The location of the tumor also played a pivotal role in the success of SLN mapping. Supraglottic tumors demonstrated significantly better outcomes, with detection rates of 93.7% and a sensitivity of 96%, compared to 84.7% and 71%, respectively, for transglottic tumors. This finding underscores the anatomical differences between these regions, which likely influence lymphatic drainage patterns and accessibility of sentinel nodes. Complex lymphatic drainage in the supraglottic region allows for the undetected spread to regional lymph nodes. Furthermore, supraglottic tumors are often more advanced and histologically aggressive than glottic tumors, even at early stages [52]. Clinicians should take tumor location into account when planning SLNB, as it may directly impact the diagnostic yield of the procedure.

Evaluation of publication bias revealed some asymmetry in the funnel plots for detection rate and sensitivity pooling. While the trim and fill method showed only a modest 4% reduction in the pooled detection rate, the adjusted sensitivity was reduced by 8%, indicating that publication bias could be a greater concern for sensitivity estimates. These findings suggest caution when interpreting sensitivity results, as smaller or less rigorous studies may disproportionately influence pooled estimates.

## 5. Conclusions

Overall, sentinel lymph node mapping demonstrates high diagnostic accuracy and reliability in staging cervical lymph nodes in laryngeal SCC, particularly when radiotracer techniques are employed. The technique is most effective in patients with supraglottic tumors, where the highest detection rates and sensitivity are observed. These findings support the adoption of SLNB as a viable alternative to more invasive staging procedures and provide valuable insights for optimizing its implementation in clinical practice.

## Figures and Tables

**Table 1:** Characteristics of the included articles.

First Author/Year	Country	Number of Patients			Mapping Material	Tracer Dosage	Wait Time After Injection	Detection Rate		Sensitivity		SLN Bilaterality	Gold Standard
		Total	Supraglottic	Transglottic				Supraglottic	Transglottic	Supraglottic	Transglottic		
Alex [25] 2000	USA	4	3	1	Radiotracer: peritumoral	0.1 mCi ^99m^Tc-sulfur colloid in 1–2.5 mL, intradermal injection	30 min	100%	100%	100%	N/A	3	Neck dissection
Fang [26] 2001	China	23	14	9	Blue dye: Submucosal injection	2% methylene blue (total volume: 2–3 mL) injected submucosally at four points: 0.5 mL administered at each site, located approximately 0.5 cm above, below, left, and right of the tumor	15–30 min	100%	88%	100%	N/A	4 supraglottoc	Neck dissection
Werner [27] 2002	Germany	15	10	5	Radiotracer: peritumoral	1.2 mCi ^99m^Tc-nanocolloid in 0.2–0.35 mL saline, injected at four sites	2–6 h	100%	100%	100%	100%	None	Neck dissection
Werner [28] 2004	Germany	31	19	12	Radiotracer: peritumoral	1.2 mCi ^99m^Tc-nanocolloid in 0.2–0.35 mL saline, injected at four sites	15–20 min	100%	100%	100%	67%	8 (7 epiglottis and 1 glotic)	Neck dissection
Werner [29] 2005	Germany	11	11	0	Radiotracer: peritumoral	45 MBq ^99m^Tc-nanocolloid in 0.2–0.35 mL saline, injected at four sites	2–6 h	100%	N/A	100%	N/A	6	Neck dissection
Hoft [30] 2004	Germany	12	N/A	N/A	Radiotracer: peritumoral	50 MBq ^99m^Tc-colloid in 0.2 mL saline, injected into at least four sites	On the day of surgery	N/A	N/A	N/A	N/A	2	Neck dissection
López Mollá [31] 2006	Spain	19	13	6	Radiotracer: peritumoral	1 mCi ^99m^Tc-human serum albumin radiocolloid, injected into four sites	1 h before surgery	92%	83%	75%	0%	4	Neck dissection
Prgomet [32] 2008	Croatia	14	9	5	Radiotracer: peritumoral	0.5 mCi/0.4 mL ^99m^Tc-human serum albumin radiocolloid	3 h before surgery	100%	83%	75%	N/A	2	Neck dissection
Chone [33] 2008	Brazil	5	N/A	N/A	Radiotracer: peritumoral	12.8 MBq ^99m^Tc-colloidal human serum albumin in 0.5 mL isotonic sodium chloride, submucosal injection	2 h before surgery	N/A	100%	N/A	N/A	N/A	Neck dissection
Tomifuji [34] 2008	Japan	20	6	14	Radiotracer: peritumoral	0.2 mL ^99m^Tc-phytate (74 MBq/mL), submucosal injection at 3 to 4 sites	The day before surgery	100%	100%	100%	50%	8	Neck dissection
Li [35] 2009	China	33	18	15	Radiotracer: peritumoral	0.15 mCi ^99m^Tc-sulfur colloid, submucosal injection at 3 to 4 points	10–12 h	94%	87%	100%	75%	6	Neck dissection
Lawson [36] 2010	Belgium	29	29	0	Radiotracer: peritumoral	1 mCi ^99m^Tc-albumin nanocolloid in 1 mL saline, submucosal injection at five sites	1–2 h	100%	N/A	100%	N/A	14	Neck dissection
Yoshimoto [37] 2012	Japan	16	N/A	N/A	Radiotracer: peritumoral	35–592 MBq/mL ^99m^Tc-labeled phytate in 0.2–0.8 mL, submucosal injection at four sites	The day before surgery or on the day of surgery	N/A	N/A	N/A	N/A	N/A	Neck dissection
Flach [38] 2013	Netherlands	13	3	10	Radiotracer: peritumoral	40 MBq ^99m^Tc-nanocolloid in 0.4 mL, injected at four sites	5–6 h post-injection	100%	90%	100%	67%	3	Neck dissection
Cheng [39] 2010	China	40	22	18	Radiotracer: peritumoral **^1^**	28–44 MBq ^99m^Tc-sulfur colloid (0.2–0.5 mL), submucosal injection at 2 to 4 points	16 h before surgery	91%	83%	89%	67%	N/A	Neck dissection
Zhao [40] 2014	China	33	10	23	Radiotracer and blue dye: peritumoral	0.15 mCi ^99m^Tc-sulfur colloid and 0.5 mL methylene blue (1%), injected at four points	On the day of surgery	90%	91%	100%	83%	N/A	Neck dissection
Akyildiz [41] 2017	Turkey	18	10	8	Radiotracer: peri-epiglottic	^99m^Tc-nanocolloid	24 h before operation	100%	100%	100%	100%	10	Neck dissection
Araki [42] 2019	Japan	4	4	0	ICG	2.0 mL of 2.5 mg/mL ICG, submucosal injection at four points	Before surgery	100%	N/A	N/A	N/A	0	Follow up
Sahafi [12] 2024	Iran	26	7	19	Radiotracer: peritumoral	2 mCi/0.4 mL ^99m^Tc-phytate, submucosal injection at four sites	10 min	100%	53%	100%	67%	11	Neck dissection

**^1^** Blue dye was also injected during surgery. SLN: sentinel lymph node; ICG: indocyanine green; ^99m^Tc: technetium-99m; MBq: megabecquerel (1 mCi = 37 MBq); mCi: Millicurie; N/A: Data not available or not reported.

**Table 2:** Quality and bias assessment of the included studies.

Studies	Patient Spectrum: Selection Regardless of T Stage (Addresses Spectrum Bias)	Consecutive Recruitment: Mitigates Selection Bias	Gold Standard Applied to All Patients	Blinded Application of Gold Standard (Addresses Partial and Differential Verification Biases)
Alex et al. [25] 2000	Yes	Consecutive	Neck dissection	Yes
Fang et al. [26] 2001	Yes	N/A	Neck dissection	Yes
Werner et al. [27] 2002	No	N/A	Neck dissection	Yes
Werner et al. [28] 2004	N/A	N/A	Neck dissection	Yes
Werner et al. [29] 2005	No	N/A	Neck dissection	Yes
Hoft et al. [30] 2004	No	N/A	Neck dissection	Yes
López Mollá et al. [31] 2006	Yes	N/A	Neck dissection	Yes
Prgomet et al. [32] 2008	No	N/A	Neck dissection	Yes
Chone et al. [33] 2008	No	Consecutive	Neck dissection	Yes
Tomifuji et al. [34] 2008	No	Consecutive	Neck dissection	Yes
Li et al. [35] 2009	Yes	N/A	Neck dissection	Yes
Lawson et al. [36] 2010	No	Consecutive	Neck dissection	Yes
Yoshimoto et al. [37] 2012	N/A	N/A	Neck dissection	Yes
Flach et al. [38] 2013	No	N/A	Neck dissection	Yes
Cheng et al. [39] 2010	Yes	N/A	Neck dissection	Yes
Zhao et al. [40] 2014	Yes	N/A	Neck dissection	Yes
Akyildiz et al. [41] 2017	No	N/A	Neck dissection	Yes
Araki et al. [42] 2019	N/A	N/A	Follow up	No
Sahafi et al. [12] 2024	No	N/A	Neck dissection	Yes

## References

[1] Sanabria A., Shah J.P., Medina J.E., Olsen K.D., Robbins K.T., Silver C.E., Rodrigo J.P., Suárez C., Coca-Pelaz A., Shaha A.R. (2020). Incidence of occult lymph node metastasis in primary larynx squamous cell carcinoma, by subsite, T classification and neck level: A systematic review. Cancers.

[2] Cho J.H., Lee Y.S., Sun D.I., Kim M.S., Cho K.J., Nam I.C., Kim C.S., Kim S.Y., Park Y.H., Joo Y.H. (2016). Prognostic impact of lymph node micrometastasis in oral and oropharyngeal squamous cell carcinomas. Head Neck.

[3] Ferlito A., Rinaldo A. (2000). Controversies in the treatment of N0 neck in laryngeal cancer: Neck dissection, no surgery or sentinel lymph node biopsy?. ORL J. Oto-Rhino-Laryngol. Its Relat. Spec..

[4] Bradford C.R., Ferlito A., Devaney K.O., Mäkitie A.A., Rinaldo A. (2020). Prognostic factors in laryngeal squamous cell carcinoma. Laryngoscope Investig. Otolaryngol..

[5] Petrakos I., Kontzoglou K., Nikolopoulos T., Papadopoulos O., Kostakis A. (2012). Glottic and supraglottic laryngeal cancer: Epidemiology, treatment patterns and survival in 164 patients. J. Buon.

[6] Sharbel D.D., Abkemeier M., Groves M.W., Albergotti W.G., Byrd J.K., Reyes-Gelves C. (2021). Occult metastasis in laryngeal squamous cell carcinoma: A systematic review and meta-analysis. Ann. Otol. Rhinol. Laryngol..

[7] Salzano G., Perri F., Maglitto F., Togo G., De Fazio G.R., Apolito M., Calabria F., Laface C., Vaira L.A., Committeri U. (2021). Pre-treatment neutrophil-to-lymphocyte and platelet-to-lymphocyte ratios as predictors of occult cervical metastasis in clinically negative neck supraglottic and glottic cancer. J. Pers. Med..

[8] Bayır Ö., Toptaş G., Saylam G., İzgi T.C., Han Ü., Keseroğlu K., Akyıldız İ., Korkmaz M.H. (2022). Occult lymph node metastasis in patients with laryngeal cancer and relevant predicting factors: A single-center experience. Tumori J..

[9] Bocca E., Calearo C., Vincentiis I.D., Marullo T., Motta G., Ottaviani A. (1984). Occult metastases in cancer of the larynx and their relationship to clinical and histological aspects of the primary tumor: A Four-Year multicentric research. Laryngoscope.

[10] Ma H., Lian M., Feng L., Li P., Hou L., Liu H., Chen X., Huang Z., Fang J. (2014). Management of cervical lymph nodes for cN0 advanced glottic laryngeal carcinoma and its long-term results. Acta Oto-Laryngol..

[11] Kim S.-J., Pak K., Kim K. (2019). Diagnostic accuracy of F-18 FDG PET or PET/CT for detection of lymph node metastasis in clinically node negative head and neck cancer patients; A systematic review and meta-analysis. Am. J. Otolaryngol..

[12] Sahafi P., Saber Tanha A., Daghighi M., Khadivi E., Khazaeni K., Vahid Reza D.K., Sadeghi R. (2024). Intra-operative lymphatic mapping and sentinel node biopsy in laryngeal carcinoma using radiotracer injection. Ann. Nucl. Med..

[13] Sadeghi R., Gholami H., Zakavi S.R., Kakhki V.R.D., Horenblas S. (2012). Accuracy of 18F-FDG PET/CT for diagnosing inguinal lymph node involvement in penile squamous cell carcinoma: Systematic review and meta-analysis of the literature. Clin. Nucl. Med..

[14] Sadeghi R., Adinehpoor Z., Maleki M., Fallahi B., Giovanella L., Treglia G. (2014). Prognostic significance of sentinel lymph node mapping in Merkel cell carcinoma: Systematic review and meta-analysis of prognostic studies. BioMed Res. Int..

[15] Krag D.N., Weaver D.L. (2002). Pathological and molecular assessment of sentinel lymph nodes in solid tumors. Semins Oncol.

[16] van den Bosch S., Czerwinski M., Govers T., Takes R.P., de Bree R., Al-Mamgani A., Hannink G., Kaanders J.H. (2022). Diagnostic test accuracy of sentinel lymph node biopsy in squamous cell carcinoma of the oropharynx, larynx, and hypopharynx: A systematic review and meta-analysis. Head Neck.

[17] Joshi V.M., Wadhwa V., Mukherji S.K. (2012). Imaging in laryngeal cancers. Indian J. Radiol. Imaging.

[18] Page M.J., McKenzie J.E., Bossuyt P.M., Boutron I., Hoffmann T.C., Mulrow C.D., Shamseer L., Tetzlaff J.M., Akl E.A., Brennan S.E. (2021). The PRISMA 2020 statement: An updated guideline for reporting systematic reviews. BMJ.

[19] 1Diagnostic Accuracy Studies. https://www.cebm.ox.ac.uk/files/ebm-tools/diagnosticaccuracystudies1.pdf.

[20] DerSimonian R., Laird N. (1986). Meta-analysis in clinical trials. Control. Clin. Trials.

[21] Higgins J.P., Thompson S.G. (2002). Quantifying heterogeneity in a meta-analysis. Stat. Med..

[22] Egger M., Smith G.D., Schneider M., Minder C. (1997). Bias in meta-analysis detected by a simple, graphical test. Bmj.

[23] Duval S., Tweedie R. (2000). A nonparametric “trim and fill” method of accounting for publication bias in meta-analysis. J. Am. Stat. Assoc..

[24] Zamora J., Abraira V., Muriel A., Khan K., Coomarasamy A. (2006). Meta-DiSc: A software for meta-analysis of test accuracy data. BMC Med. Res. Methodol..

[25] Alex J.C., Sasaki C.T., Krag D.N., Wenig B., Pyle P.B. (2000). Sentinel lymph node radiolocalization in head and neck squamous cell carcinoma. Laryngoscope.

[26] Fang J., Wei X., Li S., Wang C., Tian A., Tao Y., Sun X., Zou S., Li M., Cai S. (2001). Clinical study of the sentinel lymph node of patients with laryngeal and hypopharyngeal carcinomas. Zhonghua Er Bi Yan Hou Ke Za Zhi.

[27] Werner J., Dünne A., Ramaswamy A., Folz B., Lippert B., Moll R., Behr T. (2002). Sentinel node detection in N0 cancer of the pharynx and larynx. Br. J. Cancer.

[28] Werner J.A., Dünne A.A., Ramaswamy A., Dalchow C., Behr T., Moll R., Folz B.J., Davis R.K. (2004). The sentinel node concept in head and neck cancer: Solution for the controversies in the N0 neck?. Head Neck J. Sci. Spec. Head Neck.

[29] Werner J.A., Dünne A., Davis R. (2005). Intraoperative lymphatic mapping in cases of midline squamous cell carcinoma. Acta Oto-Laryngol..

[30] Höft S., Maune S., Muhle C., Brenner W., Czech N., Kampen W., Jänig U., Laudien M., Gottschlich S., Ambrosch P. (2004). Sentinel lymph-node biopsy in head and neck cancer. Br. J. Cancer.

[31] Mollá C.L., Suárez-Varela M.M., Llatas M.C., Monforte R.S., Martînez R.L., Galofre J.D. (2006). Sentinel lymph node in tumors of the larynx: Technique and results. Acta Otorrinolaringol. Esp..

[32] Prgomet D., Huić D., Mutvar A., Rakusić Z., Bilić M., Katić V. (2008). Sentinel lymphadenectomy in squamous cell carcinoma of the pharynx and larynx. Lijec. Vjesn..

[33] Chone C.T., Magalhães R.S., Etchehebere E., Camargo E., Altemani A., Crespo A.N. (2008). Predictive value of sentinel node biopsy in head and neck cancer. Acta Oto-Laryngol..

[34] Tomifuji M., Shiotani A., Fujii H., Araki K., Saito K., Inagaki K., Mukai M., Kitagawa Y., Ogawa K. (2008). Sentinel node concept in clinically n0 laryngeal and hypopharyngeal cancer. Ann. Surg. Oncol..

[35] Li Z., Hu G., Lei C., Zhong S., Li B., Hong S., Zhu J. (2009). Localization of sentinel lymph node with radionuclide in clinically N0 laryngeal and hypopharyngeal cancers. Zhonghua Er Bi Yan Hou Tou Jing Wai Ke Za Zhi= Chin. J. Otorhinolaryngol. Head Neck Surg..

[36] Lawson G., Matar N., Nollevaux M.C., Jamart J., Krug B., Delos M., Remacle M., Borght T.V. (2010). Reliability of sentinel node technique in the treatment of N0 supraglottic laryngeal cancer. Laryngoscope.

[37] Yoshimoto S., Hasegawa Y., Matsuzuka T., Shiotani A., Takahashi K., Kohno N., Yoshida T., Kitano H. (2012). Sentinel node biopsy for oral and laryngopharyngeal squamous cell carcinoma: A retrospective study of 177 patients in Japan. Auris Nasus Larynx.

[38] Flach G.B., Bloemena E., van Schie A., Hoekstra O.S., van Weert S., Leemans C.R., de Bree R. (2013). Sentinel node identification in laryngeal cancer: Feasible in primary cancer with previously untreated neck. Oral Oncol..

[39] Cheng Y., Wang B., Li S., Wen S., Xia L., Li X., Zhao D. (2010). A comparative study of sentinel lymph node detection in laryngeal and hypopharyngeal carcinoma by lymphoscintigraphy method and blue dye. Zhonghua Er Bi Yan Hou Tou Jing Wai Ke Za Zhi = Chin. J. Otorhinolaryngol. Head Neck Surg..

[40] Zhao X., Xiao D., Ni J., Zhu G., Yuan Y., Xu T., Zhang Y. (2014). The clinical value of sentinel lymph node detection in laryngeal and hypopharyngeal carcinoma patients with clinically negative neck by methylene blue method and radiolabeled tracer method. Lin Chuang Er Bi Yan Hou Tou Jing Wai Ke Za Zhi = J. Clin. Otorhinolaryngol. Head Neck Surg..

[41] Akyildiz S., Kaya I., Ozturk K., Turhal G., Yavuzer A. (2017). Effectiveness of sentinel lymph node biopsy with pre-epiglottic Tc99 injection. B-Ent.

[42] Araki K., Tomifuji M., Shiotani A., Hirano S., Yokoyama J., Tsukahara K., Homma A., Yoshimoto S., Hasegawa Y. (2020). Minimally invasive surgery for laryngopharyngeal cancer: Multicenter feasibility study of a combination strategy involving transoral surgery and real-time indocyanine green fluorescence-navigated sentinel node navigation surgery. Head Neck.

[43] Hassanzade M., Attaran M., Treglia G., Yousefi Z., Sadeghi R. (2013). Lymphatic mapping and sentinel node biopsy in squamous cell carcinoma of the vulva: Systematic review and meta-analysis of the literature. Gynecol. Oncol..

[44] Mehrabi B.M., Forghani M.N., Memar B., Jangjou A., Dabbagh K.V., Zakavi S.R., Aryana K., Abd Elahi A., Sadeghi R. (2010). Sentinel lymph node biopsy in melanoma patients: An experience with Tc-99m antimony sulfide colloid. Iran J. Nucl. Med..

[45] Cody H.S. (1999). Sentinel lymph node mapping in breast cancer. Breast Cancer.

[46] Hassanzadeh M., Hosseini Farahabadi E., Yousefi Z., Kadkhodayan S., Zarifmahmoudi L., Sadeghi R. (2016). Lymphatic mapping and sentinel node biopsy in ovarian tumors: A study using intra-operative Tc-99m-Phytate and lymphoscintigraphy imaging. J. Ovarian Res..

[47] Zarifmahmoudi L., Ghorbani H., Sadri K., Tavakkoli M., Keshvari M., Salehi M., Sadeghi R. (2019). Sentinel node biopsy in urothelial carcinoma of the bladder: Systematic review and meta-analysis. Urol. Int..

[48] Deganello A., Ruaro A., Gualtieri T., Berretti G., Rampinelli V., Borsetto D., Russo S., Boscolo-Rizzo P., Ferrari M., Bussu F. (2023). Central Compartment Neck Dissection in Laryngeal and Hypopharyngeal Squamous Cell Carcinoma: Clinical Considerations. Cancers.

[49] Mukherji S.K., Armao D., Joshi V.M. (2001). Cervical nodal metastases in squamous cell carcinoma of the head and neck: What to expect. Head Neck J. Sci. Spec. Head Neck.

[50] Khadivi E., Daghighi M., Khazaeni K., Kakhki V.R.D., Zarifmahmoudi L., Sadeghi R. (2015). Intra-operative lymphatic mapping and sentinel node biopsy in laryngeal carcinoma: Preliminary results. Iran. J. Otorhinolaryngol..

[51] Bézu C., Coutant C., Salengro A., Daraï E., Rouzier R., Uzan S. (2011). Anaphylactic response to blue dye during sentinel lymph node biopsy. Surg. Oncol..

[52] Hirvikoski P., Virtaniemi J., Kumpulainen E., Johansson R., Kosma V.-M. (2002). Supraglottic and glottic carcinomas: Clinically and biologically distinct entities?. Eur. J. Cancer.

